# Time Spent Jogging/Running and Biological Aging in 4458 U.S. Adults: An NHANES Investigation

**DOI:** 10.3390/ijerph20196872

**Published:** 2023-10-02

**Authors:** Christina M. Blackmon, Larry A. Tucker, Bruce W. Bailey, Lance E. Davidson

**Affiliations:** Department of Exercise Sciences, College of Life Sciences, Brigham Young University, Provo, UT 84602, USA

**Keywords:** cellular aging, telomeres, physical activity, jogging, fitness, disease risk

## Abstract

Telomere length is a good index of cellular aging. Longer telomeres are predictive of longer life, and healthy lifestyles are associated with longer telomeres. This study explored the relationship between time spent jogging or running each week and leukocyte telomere length (LTL) in 4458 randomly selected U.S. adults. The association was studied using data collected by the National Health and Nutrition Examination Survey (NHANES), and a cross-sectional design. Total weekly jog/run time was calculated from survey responses. From the minute totals, three categories were formed: <10 min/week, 10–74 min/week, and ≥75 min/week. Adults in the third category met the U.S. guidelines. Data were analyzed using one-way ANOVA. Partial correlation was used to adjust for differences in potential mediating factors, including demographic and lifestyle/medical factors. In the total sample, after adjusting for all the potential covariates, mean LTL significantly differed across the three jog/run categories (F = 4.1, *p* = 0.0272). Specifically, adults who met the guidelines via jogging and/or running had significantly longer telomeres than adults who performed no jogging/running. Adults in the middle category did not differ from the other two categories. A minimum of 75 min of jogging/running weekly is predictive of longer telomeres when compared to adults who do not jog or run regularly.

## 1. Introduction

Over the past century, life expectancy in the U.S. has shown a nearly continual upward trend. Since 1900, the expected lifespan for a U.S. adult has increased by roughly 30 years [1]. The mechanisms behind the lengthened lifespan vary from improved sanitation to medical technological advancements to higher average education [2,3,4]. Still greater improvements are likely to be seen for years to come, continually increasing the length of life.

The recognition of a greater life expectancy also encourages the need for enhanced quality of life throughout the extended years lived. Compression of morbidity plays a key role in protecting high quality of life into the later years. Many factors can impact the ability to compress years of morbidity and simultaneously extend life, including genetics, smoking, diet, and exercise [5,6,7,8]. The precise mechanisms by which each of these affect morbidity risk has been heavily researched, and one of the many possible pathways of influence connected to all the factors is their impact on telomeres.

Telomeres are the end caps of chromosomes. Telomeres protect our genetic material during cellular replication and division. Through the countless replication and division cycles that occur throughout the lifespan, telomeres shorten. Thus, telomeres act as a type of biological clock, with shorter telomeres being highly associated with older age [9]. Over years of shortening, telomeres can ultimately be destroyed. This leaves the DNA vulnerable to damage and increases the risk of individuals developing age-related diseases [10,11]. Consequently, techniques and practices for preserving telomere length are a valuable area for focused research in search of solutions for lengthened quantity and quality of life.

Studies on possible factors associated with telomere length have found genetic disorders [12,13], dietary patterns [14,15], and smoking habits [16] to be predictive of telomere shortening. Additional findings agree that physical activity is also correlated with telomere length. A majority of published articles have found a direct relationship between PA and telomere length, with more activity associated with longer telomeres [17,18]. Specifically, using the current U.S. guidelines for physical activity as a defining standard, performing exercise at or above the recommended level is associated with greater telomere lengths [19]. The general guidelines of 150 min of moderate-intensity physical activity or 75 min of vigorous physical activity per week, however, leave many unanswered questions pertaining to the relationship between exercise intensity and telomere length.

Data from a self-report survey of several thousand U.S. adults indicated that 47% state that they regularly meet the aerobic physical activity recommendations [20], a percentage that may actually be less than 10% when objectively measured [21]. Of the less than 10% that meet the guidelines according to accelerometry, most meet the guidelines via moderate intensity exercise rather than vigorous [22,23]. However, several investigations indicate that the health benefits of vigorous physical activity are greater than those resulting from moderate activity [24,25]. Overall, it appears that many aspects of health, such as metabolic, cardiovascular, and mental, are improved significantly by regular vigorous physical activity [26,27,28].

Thus, simply meeting or not meeting the guidelines for exercise may not provide an adequate description of the relationship between physical activities of varying intensities and telomere length. As a result, additional investigations into the unique correlation of moderate activity as compared to vigorous activity and telomere length have been performed. Regarding vigorous physical activity and biological aging or telomere length, findings are mixed. Some studies have concluded that vigorous activity is associated with greater telomere preservation than lower intensity [29,30], while other investigations suggest that there is no meaningful difference in telomere length when comparing activities of different intensities [31,32]. Even fewer findings exist about the relationship between specific forms of physical activity and telomere length.

The most common vigorous activity in the U.S. is jogging or running [33]. To date, investigations that concentrated on jogging or running as specific forms of vigorous activity and their associations with biological aging as indicated by telomere length are almost non-existent. Hence, more research is warranted.

This study sought to determine the relationship between time spent jogging/running per week and telomere length in a U.S.-representative sample of 4458 adults. A secondary objective was to explore the degree to which the current U.S. vigorous physical activity guidelines encourage adequate levels of activity for the specific purpose of minimizing biological aging. The extent to which the jogging/running and biological aging association was influenced by nine potential confounding factors, including age, sex, race, income, BMI, diabetes status, smoking, cardiovascular disease (CVD) status, and other physical activity engagement, was also examined.

## 2. Methods

### 2.1. Study Design

The present study utilized a cross-sectional design based on data from the National Health and Nutrition Examination Survey (NHANES). This U.S. survey is conducted in two-year cycles by the U.S. Centers for Disease Control and Prevention (CDC). To ensure maximum generalizability to the U.S. population, NHANES uses a multi-stage sampling design, which begins at the level of U.S. counties, then specific roads, then distinct houses, then individuals. Oversampling of minority groups and person-level sample weightings are also utilized by NHANES for generalization purposes [34].

All participants in the survey provided written consent for their data to be used and made publicly available. NHANES data are accessible on the CDC website. Leukocyte telomere length (LTL) information was only collected in the 1999–2000 and the 2001–2002 survey cycles and made available online in 2014. Data were gathered and published with approval from the Ethics Review Board of the National Center for Health Statistics [35].

### 2.2. Sample

During the four years when LTL data were collected, adults 20 years of age or older were requested to provide a DNA sample to be analyzed. Of the 10,291 eligible participants, 76% provided a viable sample. The number of usable samples was further delimited by excluding individuals aged 85 or older. NHANES assigns an age of 85 to all participants aged 85 or older to enhance confidentiality. However, given the strong relationship between chronological age and telomere length, only individuals with correct age data were included in the present study. A total of 4458 adults with complete data were included in the analyses.

### 2.3. Measures

In the present investigation, time spent in jogging/running was used as the predictor variable and leukocyte telomere length the outcome variable. Nine potential confounding factors were incorporated into the analysis. Of these covariates, four were demographic factors (i.e., age, sex, race, and annual income) and five were lifestyle/medical factors (smoking packyears, BMI, heart disease, diabetes, and time spent in physical activities other than jogging and/or running).

#### 2.3.1. Jogging/Running

The NHANES protocol measured physical activity using self-report questionnaires. Subject participation in 48 specific activities over the past 30 days was recorded. Jogging and running were two of the 48 activities. Beyond a yes-or-no report of engagement in jogging during the past month, subjects also recorded the frequency of engagement in days and the typical duration of each bout of jogging in minutes. The same was performed for time and frequency of participation in running [36]. According to the NHANES procedure, bouts had to last a minimum of 10 min to be included.

In the current study, the frequency and duration data for jogging and running was combined. The grouping of jogging and running data was appropriate due to the overlap of MET-values assigned to each activity by NHANES. In short, it is difficult for joggers and runners to know when they have moved from jogging to running or from running to jogging. The activities are similar. Additionally, there was no statistical difference in the length of telomeres between joggers and runners. Consequently, total combined minutes spent jogging and/or running was determined.

Identification of the aggregate time spent in jogging/running in minutes per week enabled comparison of participant activity levels to the current U.S. guidelines for vigorous physical activity. Participants engaging in the recommended 75 min or more of jogging/running per week were classified as “joggers/runners”. Individuals performing some jogging/running but not meeting the guidelines were labeled “insufficient joggers/runners”, while individuals not involved in any level of jogging/running were termed “non-joggers/runners”.

#### 2.3.2. Telomere Length

As the outcome variable of the present investigation, telomere length is a gauge of biological age. Telomeres of various tissue cells can be used, but blood cell (leukocyte) telomeres are most common because they are the best representation of all other telomeres in the body [37]. Consequently, NHANES used leukocytes to index telomere length.

The measurement procedure used by NHANES to determine the length of telomeres is detailed and precise. Care was taken during the measurement process to ensure that the margin of error was minimized. NHANES indicates that the telomere length assay was performed using the quantitative polymerase chain reaction method. Telomere length was assessed relative to standard reference DNA (T/S ratio), as described in detail elsewhere [38,39]. NHANES states, “each sample was assayed 3 times on 3 different days. The samples were assayed on duplicate wells, resulting in 6 data points. Sample plates were assayed in groups of 3 plates, and no 2 plates were grouped together more than once. Each assay plate contained 96 control wells with 8 control DNA samples. Assay runs with 8 or more invalid control wells were excluded from further analysis (<1% of runs). Control DNA values were used to normalize between-run variability. Runs with more than 4 control DNA values falling outside 2.5 standard deviations from the mean for all assay runs were excluded from further analysis (<6% of runs). For each sample, any potential outliers were identified and excluded from the calculations (<2% of samples). The mean and standard deviation of the T/S ratio were then calculated normally. The interassay coefficient of variation was 6.5%” [40]. The number of base-pairs within a telomere was determined using the formula: 3274 + 2413 × (T/S).

#### 2.3.3. Covariates

The current study utilized four demographic covariates collected by NHANES: age, sex, race/ethnicity, and household income. Age was restricted to 20–84 years, inclusive, excluding participants 85 and older. Sex consisted of two categories, male or female. The variable of race/ethnicity was determined through questions about race and Hispanic origin. The five race/ethnicity categories used in this study were Non-Hispanic White, Non-Hispanic Black, Mexican American, Other or Multi-Racial, and Other Hispanic. Household annual income was self-reported. Participants were asked to report their household income based on consecutive categories. Income categories were then collapsed to form five representative groups: USD 0–24,999; USD 25,000–44,999; USD 45,000–64,999; USD 65,000 or more; or missing.

Five lifestyle or medical covariates were also included. Body Mass Index (BMI) was calculated using the validated formula: body mass (kg) divided by height (m) squared. Individuals were then grouped according to the standard categories for BMI [41]. Diabetes status was affirmed if fasting blood glucose levels were 126 mg/dL or above, if participants reported a physician’s diagnosis of diabetes, or if they were currently taking diabetic medication. Individuals were categorized as having or not having diabetes [42]. A dichotomous normal or CVD-present variable was also included in analyses. If participants had ever experienced a heart attack, a stroke, or had been diagnosed with coronary heart disease, they were considered to have CVD [43]. Smoking pack years was recorded as a continuous variable. The calculation involved multiplying the number of cigarettes smoked per day by the number of years the participant had smoked. That number was then divided by the typical number of cigarettes in a single pack (20) [44].

The final lifestyle factor that was included in the present study was total MET-minutes spent in physical activity besides jogging/running. A MET is a metabolic equivalent, and one MET equals the resting energy expenditure of an individual [45]. For each of the 48 surveyed activities, subjects provided three descriptions for each of the activities they participated in at least once for a minimum of 10 min in the last 30 days: the frequency of participation in total days, the average duration of each bout in minutes, and the estimated intensity level of the exercise. Intensity was reported as either moderate or vigorous according to descriptions provided to the subjects by NHANES. Specific MET levels were assigned to the moderate and vigorous intensity delineations of each activity. From these data, total MET-minutes were calculated by multiplying the frequency, duration, and MET intensity values for each activity and summing the values for all the activities. Jogging/running MET-minutes were then subtracted from the total to obtain a value of all MET-minutes of physical activity, excluding time spent in jogging/running.

### 2.4. Statistical Analysis

As a result of the sampling process employed by NHANES, sample weights were assigned to each subject. The weightings enabled the data to be generalized to all non-institutionalized U.S. adults. The sample weights provided a correction for the unequal selection probability, nonresponse distortions, and independent population controls [46].

Typically, statistical power is based primarily on the number of participants in the sample. Given the large sample size of the present investigation, power would be expected to be excellent. However, given the multi-level sampling strategy used by NHANES, statistical power was reduced substantially. Specifically, degrees of freedom in the denominator were calculated by subtracting the number of strata (28) from the number of clusters (57), not the number of participants. Therefore, the number of degrees of freedom in the study was only 29 rather than several thousand.

One-way analysis of variance (ANOVA) using multiple regression (SurveyReg) was employed to determine the extent to which mean telomere length differed across the jogging/running categories based on meeting the U.S. physical activity guidelines. Potential confounding variables were controlled statistically using partial correlation. Telomere length means were adjusted for differences in the covariates (age, sex, race, income, BMI, diabetes status, smoking pack years, CVD status, and time spent participating in physical activities other than jogging/running) using the LSmeans procedure. Statistical differences were considered significant when *p* < 0.05. SAS software (version 9.4) was used to analyze the NHANES data.

## 3. Results

The analysis is based on a U.S.-representative sample of 4458 adults, including both women and men. Table 1 displays the means and percentiles of the continuous variables included in the analysis. All adults were 20–84 years old with a sample mean (±SE) age of 42.3 ± 0.4 years. The mean telomere length of the sample was 5896 ± 40 base pairs, and the average weekly minutes spent jogging/running was 8.1 ± 1.0 for the entire sample. Among adults reporting at least 10 min of jogging/running per week, the mean (±SE) minutes per week was 91.5 ± 9.4. The frequency and sample percentages for the categorical variables are shown in Table 2.

A significant bivariate, linear relationship was observed between age and telomere length (F = 124.1, *p* < 0.0001, r = −0.32). From the regression coefficient, it was determined that for every one-year increase in chronological age, telomeres were 15.4 base pairs shorter, on average.

As shown in Table 3, after controlling for the demographic covariates, including age, sex, race, and income (Model 1), mean telomere lengths differed significantly across the categories of jogging/running time (F = 6.5, *p* = 0.0046). Specifically, adults who jogged/ran 75 min per week or more, those who met the U.S. physical activity guidelines and were in the highest jogging/running category, had significantly longer telomeres than individuals who did not engage in regular jogging/running. The difference was 216 base pairs. Differences in LTL between those in the non-joggers/runners category (lowest category) and those in the joggers/runners (highest category) did not differ significantly from those in the insufficient joggers/runners category (middle category).

Expanding the demographic model to include lifestyle/medical covariates (smoking packyears, BMI, diabetes status, MET-minutes of physical activity other than jogging/running, and heart disease status), weekly jogging/running time remained a significant predictor of telomere length (F = 4.1, *p* = 0.0272). Particularly, those who met the U.S. guidelines of 75 min of vigorous activity through jogging/running time per week were noted to have telomeres that were, on average, 189 base pairs longer than those in the no jogging/running category. No other mean telomere lengths were statistically different when comparing the three levels of participation in jogging/running.

## 4. Discussion

The primary purpose of this study was to determine the association between weekly jog/run time and leukocyte telomere length, a key indicator of cellular aging, in a nationally representative sample of U.S. adults. Additionally, the study aimed to evaluate the alignment of the current U.S. vigorous physical activity guidelines to telomere health. Differences in various demographic and lifestyle factors were controlled prior to analysis of the associations to limit potential confounding influences.

Findings from the study revealed a significant relationship between time spent jogging/running and telomere length—those who engaged in a minimum of 75 weekly minutes of jogging/running presented with longer telomeres, on average, than those who reported no jogging/running per week (<10 min of jogging/running per week). The relationship, however, was not dose–response. Those who participated in some jogging/running, but did not meet the U.S. physical activity guidelines, did not have LTLs that differed significantly from the no jogging/running group or the high jogging/running group. This lack of significance may indicate the need for further investigation into other factors that could play a moderating role in the relationship. Additionally, it could mean that jogging or running less than 75 min per week is simply not enough work to protect leukocyte telomeres and slow biological aging. Nonetheless, it appears that 75 min of vigorous activity per week achieved via jogging and/or running is a valid recommendation due to the finding that adults in this category had significantly longer telomeres than adults who did not engage in jogging or running.

Controlling for the demographic and lifestyle/medical covariates marginally decreased the significance of the relationship, but significance was maintained. Average telomere length in the no jogging/running group after controlling for the lifestyle/medical factors was nearly identical to controlling for the demographic factors alone. A shortening in average LTL was observed, however, between Model 1 and Model 2 in both the middle group, those that performed between 10–74 min of jogging/running per week, and the upper group, those jogging/running at least 75 min weekly. This observation may be the result of the individuals in the no jogging/running group being similar in terms of lifestyle and medical risk factors, while those in the upper two groups having greater variation regarding health status and lifestyle. Nonetheless, a comparison of both models indicates that, even if all participants had the same demographic, lifestyle, and medical factors, individuals meeting the U.S. vigorous activity guidelines through jogging/running have, on average, longer telomeres than those who do not engage in jogging/running.

Previous studies exploring the relationship between general engagement in physical activity and telomere length have formed the foundation for the present investigation. One of the earliest studies performed in this area measured the relationship between self-reported physical activity level on a 4-point scale and leukocyte telomere length. The conclusion was that higher overall participation in physical activity is linked to longer telomeres, with the most active participants having telomeres that were, on average, 200 base pairs longer than the least active participants [47]. Tucker later published a supporting conclusion after utilizing NHANES data to identify a direct positive relationship between total physical activity involvement and telomere length, reporting a 7-year aging advantage from regular activity engagement compared to inactivity [19]. Similar findings were also reported in studies performed on all-women samples, noting that, regardless of age, women who engaged in higher amounts of leisure-time physical activity had reduced biological aging, indicated by the preservation of telomere length [48,49]. General trends from the current literature indicate a direct relationship between general engagement in physical activity and LTL.

Looking more closely at the physical activity and telomere length association, the body of research focusing on specific types of physical activity or specific intensities in relation to telomere attrition is limited. However, some studies have explored this relationship at some level. For example, secondary analyses from the aforementioned studies by Du et al. and Shadyab et al. found that women who engaged in activities with intensities of 3 METs or greater tended to have longer telomeres than those who were only active at intensities below 3 METs [48,49]. Additionally, two studies performed on endurance-trained (running and/or cycling) athletes reported longer telomeres in the athletes than the untrained controls [50,51]. Yet, this finding contains some uncertainty, because a similar study in marathon runners failed to find any significant difference between LTL in the marathoners and the age-matched controls [52]. It is noteworthy, however, that there were only 32 participants in the marathon study. Thus, it may have lacked sufficient statistical power to identify a significant relationship between telomere length and endurance running. Nonetheless, it is clear that current findings lack consistency and, thus, a better understanding of the influence of exercise intensity on the relationship between physical activity and telomere length is desired.

No study has yet explored the specific aging advantage associated with total time spent in jogging/running in particular. Further, the value of the current U.S. activity guidelines in connection with biological aging has not been addressed in the published literature. While a 2016 study that explored the possibility of a connection between involvement in nine different activities and telomere length did conclude that running was the only exercise studied that correlated with longer telomeres, the authors recorded activity involvement as binary, not tiered. Specifically, the authors examined if subjects ran or not. Additionally, they reported being unable to analyze single-mode associations, so the results were the combined effect of all exercises in which an individual engaged [53]. Thus, there is value in more closely exploring the relationship between time spent in jogging/running and LTL as well as the relevance of the U.S. vigorous activity guidelines of 75 min or more per week in this relationship.

Using regression analyses, the study at hand identified shorter telomeres by 15.6 base pairs for each advancing year of chronological age. In other words, every one-year increase in age was correlated with telomeres that were 15.6 base pairs shorter, on average, than the previous year, after controlling for numerous potentially confounding variables. For example, 80-year-olds tended to have telomeres that were roughly 234 base pairs shorter than 65-year-olds (15 yrs × 15.6 base pairs), and 50-year-olds tended to have telomeres that were roughly 234 base pairs shorter than 35-year-olds (15 yrs × 15.6 base pairs), matched for several demographic and lifestyle/medical factors. Hence, the telomere lengths of adults differing by a couple hundred base pairs is quite substantial—as substantial as the biological aging difference between 80-year-olds and 65-year-olds, and the biological aging difference between 50-year-olds and 35-year-olds.

The present results revealed a difference in telomere length of 190 base pairs between the group that performed no jogging or running and the group that met the 75 min guideline for jogging/running per week. This variance represents a difference of approximately 12.2 years (190 ÷ 15.6) in biological age. In short, this equates to roughly a 12-year cellular aging advantage associated with jogging/running.

Reduced telomere length has been shown to correlate with increased mortality and the risk of various chronic diseases. Rode et al. performed a prospective cohort study with more than 64,600 participants observing the correlation between telomere length and mortality risk. Comparing participants in the lowest decile (shortest telomeres) to those in the highest decile (longest telomeres), they reported that risk of all-cause mortality was 1.4 times higher for those in the lowest decile of telomere length. A similar association was also documented for both CVD- and cancer-specific mortality [54]. Another study with related objectives concluded that the risk of all-cause mortality was 72% higher for participants in the lowest tertile for telomere length than for those in the highest tertile [55]. Several studies have also assessed disease prevalence or risk associated with shorter telomeres and have found greater incidence of myocardial infarction, stroke, type-2 diabetes, coronary artery disease, and other health risks in individuals with reduced telomere lengths [56,57].

Given the investigations mentioned above, the longer telomere length observed in joggers/runners compared to non-joggers/runners in this study suggests possible reduced risk of mortality and disease for the joggers and runners. But what mechanisms can explain the relationship observed between jogging/running and preserved telomere length? There is still much that is unknown about this interaction. The most compelling factor, however, may be the influence of physical activity on oxidative stress within the body. The overproduction of reactive oxygen species (usually during oxygen metabolism) can cause irreversible damage to cell tissues and DNA. The published literature has indicated clearly that such damage is highly linked to cellular aging [58,59,60]. Ancillary studies have also found chronic (though not acute) physical activity to control or reduce oxidative stress throughout the body. Several pathways have been identified by which exercise may successfully manage oxidative stress. First, regular physical activity appears to enhance the body’s antioxidant defense systems, such as activation of protective genes and greater production of DNA-repairing enzymes [17,61,62,63,64]. Additionally, consistent physical activity has been reported to reduce the presence and/or impact of reactive species and the levels of inflammatory hormones (molecules known to cause wider spread damage) [17,47,65,66].

An additional pathway by which physical activity may aid in resisting telomere attrition is through the production of irisin. Irisin is a hormone directly related to telomere length [67] that has been found to increase with physical work, like that performed in exercise [68]. Further, levels of this hormone appear to have a dose–response association with activity; as exercise intensity increases, irisin levels increase, thereby increasing the chances of telomere preservation [69,70]. This could be a key mechanism by which the enhanced biological advantage from jogging/running occurs.

We acknowledge several limitations of the current study. First, this investigation utilized a cross-sectional design. Thus, causation cannot be concluded due to the lack of temporality. Second, the physical activity data was self-reported. Self-report data are vulnerable to inaccuracies. Third, while many potentially confounding variables were controlled in this study, there are likely others that could be influencing the relationship between jogging/running and telomere length. Finally, the jogging and running variables were not analyzed separately. Both are considered vigorous activities and they overlap in their MET-intensity values, making it difficult for participants to differentiate between the activities. With an understanding of these weaknesses, future studies exploring this relationship should utilize a prospective design to gain better control of external influences on the association and allow for improved establishment of a causal relationship.

Despite limitations, the study also maintained a number of strengths. The use of NHANES data supplied a large sample size of randomly selected, U.S.-representative participants. This provided an extraordinary ability to generalize the findings to U.S. adults aged 20–84. Additionally, the measurement of leukocyte telomeres was completed in a reputable lab following well-established procedures for biological assays to determine T/S ratios and convert to base pairs. Thus, the validity of the telomere length values can be trusted. Additionally, the analyses indicated a significant correlation between chronological age and LTL, as should be observed, thereby furthering the validity of the telomere data. Finally, care was taken to isolate the association between LTL and jogging/running by adjusting for a number of key demographic and lifestyle/medical factors that were most likely to influence the relationship, including the statistical removal of time spent in physical activities other than jogging or running.

## 5. Conclusions

In a sample of 4458 U.S.-representative adults, a significant association existed between jogging/running a minimum of 75 min per week (to meet the U.S. guidelines for vigorous activity) and longer leukocyte telomeres. The leukocyte telomere length difference between the non-joggers/runners and those who met the guidelines accounted for a biological age difference of approximately 12 years in favor of the runners. This relationship was maintained following control of possible confounding demographic and lifestyle/medical factors. Clearly, more research remains to be performed to better understand the mechanisms of this association, more firmly establish a causal connection, and explore the lack of differences in telomere length for the insufficient joggers/runners. Nonetheless, the present findings support the current U.S. vigorous activity guidelines and encourage engagement in an active lifestyle through regular jogging and running at least 75 min per week.

## Figures and Tables

**Table 1:** Means, standard errors, and key percentiles for each continuous variable (*n* = 4458).

Continuous Variable	Mean	SE	10th	25th	50th	75th	90th
Age	42.3	0.4	23.7	31.1	40.9	51.8	60.9
Jog/Run min/week	8.1	1.0	0.0	0.0	0.0	0.0	0.0
Smoking pack years	3.2	0.2	0.0	0.0	0.0	0.0	10.0
Telomere length	5896	40	5188	5447	5796	6223	6724
MET-min of other exercise	778.5	63.7	0.0	0.0	0.0	691.6	2268.7

Notes: All means and percentiles were calculated using SAS. SE is standard error of the mean. “MET-min of other exercise” is the total number of MET-minutes of exercise performed per week by participants in any physical activity other than jogging and/or running.

**Table 2:** Characteristics of the sample based on the categorical variables (*n* = 4458).

Categorical Variable	N	%	SE
**Weekly Jogging/Running**			
None	4060	91.1	0.81
10–74 min	241	5.4	0.56
≥75 min	157	3.5	0.46
**Sex**			
Women	2246	50.4	0.70
Men	2212	49.6	0.70
**Race/Ethnicity**			
Non-Hispanic White	3121	70.0	2.01
Non-Hispanic Black	494	11.1	1.37
Mexican American	364	8.2	0.87
Other Race/Multiracial	160	3.6	0.61
Other Hispanic	319	7.1	1.57
**Body Mass Index**			
Normal Weight	1481	33.2	0.78
Overweight	1530	34.3	1.19
Obese	1447	32.5	1.00
**Income**			
<$25,000	1037	23.2	1.32
$25,000–44,999	862	19.3	1.07
$45,000–64,999	749	16.8	0.87
$65,000 or more	1380	31.0	1.93
Missing	430	9.7	1.08
**Diabetes Status**			
No	4126	92.6	0.53
Yes	332	7.4	0.53
**CVD Status**			
No	4245	95.2	0.42
Yes	213	4.8	0.42

Note: All frequencies and standard errors were calculated via SAS. Values in both the “N” column and the “%” column reflect the distribution of participants following application of the NHANES sample weights. This allows for generalization of the data to the U.S. population. SE provides the standard error of the reported weighted percentages. Smoking pack years and METs in other physical activities were treated as continuous variables.

**Table 3:** Differences in mean telomere lengths across levels of jogging/running per week after adjusting for the covariates.

Weekly Jogging/Running Time					
Covariate	None Mean ± SE	10–74 min Mean ± SE	≥75 min Mean ± SE	F-Value	*p*-Value
Model 1	5875 ^a^ ± 38	6005 ^a,b^ ± 91	6091 ^b^ ± 55	6.5	0.0046
Model 2	5874 ^a^ ± 38	5983 ^a,b^ ± 90	6064 ^b^ ± 61	4.1	0.0272

Note: All values were analyzed via SAS using ANOVA. Means in the same row with the same superscript (^a^ or ^b^) were not significantly different (*p* > 0.05). There were 4060 adults in the None category for jogging/running time, 241 adults in the 10–74 min category, and 157 in the ≥75 min category. Model 1 adjusted for the demographic covariates (age, sex, race/ethnicity, and annual income). Model 2 adjusted for all the variables in Model 1 plus the lifestyle/medical covariates (BMI, diabetes, heart disease, smoking pack years, and MET-minutes of other physical activity).

## Data Availability

All data supporting reported results can be found online as part of the National Health and Nutrition Examination Survey (NHANES). The data are free and can be found at the following website: https://wwwn.cdc.gov/nchs/nhanes/Default.aspx (accessed on 29 August 2023).
